# ER-Mitochondria Communication in Cells of the Innate Immune System

**DOI:** 10.3390/cells8091088

**Published:** 2019-09-15

**Authors:** Dmitry Namgaladze, Vera Khodzhaeva, Bernhard Brüne

**Affiliations:** 1 Institute of Biochemistry I, Faculty of Medicine, Goethe-University Frankfurt, 60590 Frankfurt, Germany; khojaewa.vera@mail.ru (V.K.); B.Bruene@biochem.uni-frankfurt.de (B.B.); 2 German Cancer Consortium (DKTK), Partner Site Frankfurt, 60590 Frankfurt, Germany; 3 Project Group Translational Medicine and Pharmacology TMP, Fraunhofer Institute for Molecular Biology and Applied Ecology, 60596 Frankfurt, Germany

**Keywords:** endoplasmic reticulum, mitochondria, macrophages, inflammation, lipid metabolism

## Abstract

In cells the interorganelle communication comprises vesicular and non-vesicular mechanisms. Non-vesicular material transfer predominantly takes place at regions of close organelle apposition termed membrane contact sites and is facilitated by a growing number of specialized proteins. Contacts of the endoplasmic reticulum (ER) and mitochondria are now recognized to be essential for diverse biological processes such as calcium homeostasis, phospholipid biosynthesis, apoptosis, and autophagy. In addition to these universal roles, ER-mitochondria communication serves also cell type-specific functions. In this review, we summarize the current knowledge on ER-mitochondria contacts in cells of the innate immune system, especially in macrophages. We discuss ER- mitochondria communication in the context of macrophage fatty acid metabolism linked to inflammatory and ER stress responses, its roles in apoptotic cell engulfment, activation of the inflammasome, and antiviral defense.

## 1. Introduction

Transfer of material between intracellular organelles is an essential biological signaling process of every eukaryotic cell. Interorganelle communication is executed at specialized membrane contact sites through protein-protein and protein-lipid interactions [1,2]. Among these, the communication between endoplasmic reticulum (ER) and mitochondria is the most extensively studied. It was first shown to take place at biochemically distinct entities termed mitochondria-associated membranes (MAM) [3,4], and is mechanistically as well as functionally thoroughly characterized [5,6]. Mitochondria-ER contacts have overarching roles in regulating cellular calcium homeostasis and phospholipid biosynthesis, and also participate in autophagy, mitochondrial dynamics, apoptosis, and the ER stress responses. In this review, we cover the functional roles of ER-mitochondrial communication in cells of the innate immune system, particularly macrophages and dendritic cells. Our focus is on lipid metabolism, efferocytosis, inflammasome activation, and antiviral responses. 

## 2. Basic Considerations towards the ER-Mitochondrial Communication

ER-mitochondria contacts are formed and maintained through specific protein-protein interactions, which comprises of the tethering of the two organelles and transfer of material between them. In yeast, an ER-mitochondria tethering protein complex named ER-mitochondria encounter structure (ERMES) was identified [7]. However, functional homologues of this complex in mammalian cells are still unclear, although PDZ domain-containing protein 8 (PDZD8) may be referring to the ERMES protein Mmm1 [8]. 

The first protein suggested to maintain ER-mitochondrial contacts in mammalian cells was mitofusin 2 (Mfn2) [9]. Mfn2 is present on both, the ER and mitochondrial membranes, and tethering involves Mfn2 homodimer formation. There has been a controversy regarding the effect of Mfn2 on spanning the distance between the ER and mitochondria [10,11], reflecting methodical difficulties. Mfn2 and its partner mitofusin 1 (Mfn1) also play important roles in fusion of mitochondrial outer membranes [12], but Mfn1 is apparently dispensable for the ER-mitochondria interaction. 

The list of proteins involved in ER-mitochondrial tethering is constantly growing [6]. Some of these proteins are ubiquitously expressed, whereas others may facilitate ER-mitochondrial communication in specialized, e.g. steroidogenic cells. Many of these proteins facilitate cargo transfer rather than pure tethering. 

The most recognized function of ER-mitochondrial contacts is their role in Ca^2+^ transfer from the ER to mitochondria [13,14]. The spatial proximity of inositol triphosphate receptors (IP3R) on the ER membrane and mitochondrial voltage-dependent anion channels (VDAC) allows a synaptic-like transfer of Ca^2+^ from the ER to mitochondria, thereby creating high local Ca^2+^ concentrations that provoke subsequent mitochondrial Ca^2+^ uptake through a low-affinity mitochondrial calcium uniporter (MCU) [15,16,17]. Proteins modulating ER-mitochondrial Ca^2+^ transfer include Mfn2 [9], sigma 1 receptor [18], glucose-regulated protein 75 (Grp75) [17], and polycystin 2 [19].

ER-mitochondrial contacts are crucial in regulating mitochondrial dynamics, i.e., fusion and fission [20]. Mitochondrial fragmentation, mediated by the GTPase dynamin-related protein 1 (Drp1) mostly occurs at ER-mitochondria contact sites, where ER wraps and constricts mitochondria [21]. ER-mitochondria contacts may also regulate mitochondrial DNA (mtDNA) replication, and coordinate the distribution of newly synthesized mtDNA between fissioning mitochondria [22]. Another well-established role of ER-mitochondria contacts is phospholipid transfer [23]. Initial studies on MAM showed a distinct compartmentalization of phospholipid biosynthesis, with phosphatidylserine (PS) synthase residing in MAM, whereas PS decarboxylase is located in mitochondria [24,25]. Efficient transfer of newly synthesized PS to mitochondria requires close apposition of ER and mitochondrial membranes [23]. In yeast, both ERMES and ER-membrane protein complexes participate in the ER-mitochondria phospholipid transfer [7,26]. In mammalian cells members of oxysterol-binding protein-related protein (ORP) family, ORP5 and ORP8 may contribute to PS trafficking between the ER and mitochondria [27]. Vacuolar protein sorting 13 proteins may also contribute to lipid transfer between the ER and mitochondria, as well as other organelles [28]. However, details of non-vesicular lipid transfer from ER to mitochondria in mammalian cells remain unclear for many lipid species [29]. 

ER-mitochondria contacts are also engaged in regulating apoptosis [30]. Stabilization of Drp1, via SUMOylation by a mitochondrial anchored protein ligase at ER-mitochondrial contact sites, facilitates cristae remodeling and cytochrome c release post Bax/Bak activation, likely through Ca^2+^ transfer from the ER to mitochondria [31]. ER-mitochondria contact sites apparently supply lipids for nascent autophagosome formation [32], and may provide platforms for the assembly of signaling complexes, such as mechanistic target of rapamycin complex 2 [33]. 

## 3. The Innate Immune System

The innate immune system, with its major cellular players monocytes/macrophages, dendritic cells, and neutrophils, is not only critical to combat pathogens, but also performs important homeostatic functions, such as clearance of dying cells, lipoprotein metabolism, and tissue repair. Innate immune cells, particularly macrophages, show remarkable plasticity, adopting multiple phenotypes when encountering specific stimuli of their microenvironment. These responses not only occur at the level of gene expression, but are also accompanied by metabolic changes, e.g. switching from oxidative phosphorylation to glycolysis in response to pathogens [34]. Metabolic alterations also involve inter-organelle communications, including ER-mitochondria communication. In the following paragraphs we address physiologically relevant aspects of ER-mitochondria communication in innate immune cells and touch upon lipid metabolism, apoptotic cell engulfment, inflammasome activation, and antiviral responses. 

## 4. ER-Mitochondria in Macrophage Lipid Metabolism

### 4.1. Introduction to Macrophage Lipid Metabolism 

Macrophage lipid metabolism plays important roles in organismal physiology and in the pathogenesis of major metabolic diseases such as diabetes or atherosclerosis [35]. Thus, macrophages actively engage in the metabolism of lipoproteins, taking up native as well as oxidatively modified lipoproteins via macropinocytosis or scavenger receptors. Whereas macrophages have effective mechanisms to efflux cellular cholesterol, excessive lipoprotein uptake under hyperlipidemic conditions or due to defects of cholesterol efflux causes formation of lipid-laden foam cells typical for the pathology of atherosclerosis. Excessive macrophage cholesterol accumulation triggers inflammatory responses such as inflammasome activation, whereas free cholesterol overload of the ER provokes ER stress and cell death [36]. Regarding fatty acid metabolism, macrophage fatty acid β-oxidation (FAO) is an important fuel source, contributing to cellular energy homeostasis and regulating inflammatory responses to extracellular fatty acids [37]. Macrophages are also prominent in generating oxidative derivatives of polyunsaturated fatty acids via cyclooxygenase and lipoxygenase family members, with distinct roles in propagation as well as resolution of inflammation [38,39].

### 4.2. ER Stress Response to Lipid Overload

The ER and mitochondria have multiple functions in lipid metabolism. ER is a biosynthetic hub for most complex lipids and bioactive eicosanoids, a place where nascent lipid droplets are formed for intracellular lipid storage, and plays a central regulatory role to control cellular cholesterol levels. Mitochondria also participate in phospholipid biosynthesis and provide energy for ER biosynthetic processes. Thus, proper functions of both organelles are crucial for lipid homeostasis. ER malfunction manifests as an unfolded protein response (UPR), which is controlled by three ER transmembrane sensors, i.e., inositol requiring enzyme 1 (IRE1), protein kinase RNA-activated (PKR)-like ER kinase (PERK), and activating transcription factor 6 (ATF6) [40]. IRE1 possesses endoribonuclease activity and thus, splices the transcription factor X-box binding protein 1 (XBP1). Together with ATF6, they induce expression of ER chaperones to fold enzymes and proteasome components, restoring ER homeostasis. Activated PERK phosphorylates translation initiation factor 2α (eIF2α), attenuating protein synthesis, while allowing translation of select proteins, including transcription factors ATF4 and C/EBP-homologous protein (CHOP). Whereas induction of ATF4 and CHOP also serves to restore ER homeostasis, excessive UPR may cause apoptosis. Under physiological conditions macrophages efficiently convert fatty acids and cholesterol into triglycerides and cholesteryl esters to store them in lipid droplets. Lipid overload during obesity or in the context of atherosclerotic plaques overwhelms these mechanisms casing increased delivery of saturated fatty acids (SFA) and unesterified cholesterol in the ER. Increasing saturation degree of the ER phospholipids directly activates the UPR effectors PERK and IRE1 [41]. Elevations of ER unesterified cholesterol, on the other hand, inhibit sarcoplasmic/endoplasmic reticulum calcium ATPase, causing disruption of ER Ca^2+^ homeostasis and triggering the UPR [42]. SFA elicit inflammatory responses in macrophages [43,44], and these effects contribute to the development of insulin resistance and diabetes [45]. Mechanistically, SFA activate c-Jun N-terminal kinase (JNK) and nuclear factor κB (NFκB) signaling cascades [45,46,47], with established links to UPR signaling [48,49].

### 4.3. Roles of FAO and Mitochondrial Dynamics in Inflammatory Responses to SFA 

Mitochondria regulate macrophage responses to SFA through FAO [37]. Studies from our laboratory showed that FAO attenuates inflammatory and ER stress responses in palmitate-treated THP-1 macrophages and primary human macrophages [50]. Interestingly, disrupting FAO increases palmitate incorporation into phospholipids with a mitochondrial/MAM biosynthetic location, affecting phosphatidylethanolamine and PS, whereas phosphatidylcholine fatty acid composition remained unaffected. Apparently, mitochondrial fatty acid metabolism exerts a metabolic control on ER phospholipid homeostasis, the molecular details of which remain to be investigated. It is also unclear whether altering ER-mitochondrial tethering affects fatty acid incorporation into phospholipids under conditions of fatty acid overload. It should be noted that in myeloid-specific carnitine palmitoyl transferase 2 knockout mice FAO only marginally contributed to obesity-related adipose tissue inflammation [51]. This questions the in vivo significance of macrophage FAO for lipid overload-related inflammation.

Recently, we established a connection between fatty acid metabolism and mitochondrial dynamics [52]. Fatty acids induced mitochondrial fragmentation in primary human macrophages, independently of their chain length and degree of saturation. Decreasing fragmentation aggravated inflammatory responses to SFA, probably by increasing mitochondrial reactive oxygen species (mROS) [53]. Whether fatty acid-induced mitochondrial fragmentation affects ER-mitochondrial communication in macrophages, such as Ca^2+^ fluxes, remains unexplored. Interestingly, whereas mitochondrial ROS production showed little effect on SFA-induced inflammatory signaling under standard cell culture conditions, it increased palmitate-induced inflammation by enhancing JNK activity under hypoxia, which may be of relevance for obese conditions [53]. 

### 4.4. ER-Mitochondrial Contacts Modulate Lipid Overload-Induced ER and Inflammation

JNK activation by SFA may involve Src tyrosine kinase translocation to intracellular lipid raft-like structures [54], where Src promotes activation of mixed-lineage protein kinase 3, the upstream kinase of the JNK signaling cascade [55]. Notably, MAM are enriched in cholesterol and sphingolipids as compared to bulk ER and may provide platforms for Src family kinase (SFK) activation. Several SFK are prominently expressed in myeloid cells, and although only Src was investigated in the context of obesity, other SFK were linked to inflammatory responses in macrophages. Interestingly, SFK Fgr was recently shown to activate mitochondrial complex II during bacterial infection, which contributed to antibacterial defenses [56]. While these findings highlight the significance of mitochondrial location of SFK for inflammatory signaling, the factors regulating SFK organelle targeting and whether it is controlled by altering ER-mitochondria contact sites awaits investigation.

Studies in non-immune cellular systems suggested that MAM integrity controls lipid overload-triggered UPR, contributing to the development of insulin resistance in various organs [57]. Proteins involved in the UPR, such as Bip/Grp78, IRE1, and PERK reside in MAM fractions [18,58,59,60]. Disrupting MAM through silencing of Grp75, Mfn2, or inhibiting cyclophilin D increased expression of ER stress markers in hepatoma cells and contributed to the development of hepatic insulin resistance under conditions of SFA overload [61]. However, another study showed that tightening ER-mitochondrial contacts in the liver caused ER stress and steatosis, suggesting that mitochondrial Ca^2+^ overload, through enhanced ER-mitochondrial coupling, caused mitochondrial dysfunction with consequent stress responses [62]. Thus, the exact role of ER-mitochondrial communication in hepatic metabolism remains unresolved.

Several studies explored the impact of Mfn2 on ER stress responses. Mfn2 ablation in the liver and skeletal muscle increased ER stress markers and promoted hyperlipidemia-induced insulin resistance [63]. While specific contributions of Mfn2-dependent ER-mitochondrial tethering versus mitochondrial fusion were not addressed in that study, later findings suggested that Mfn2 interacts with PERK, repressing its activity, providing a mechanistic explanation for enhanced ER stress in Mfn2-deficient cells [64]. More recent work proposed a direct influence of Mfn2 on hepatic phospholipid homeostasis [65]. Mfn2 was shown to directly bind PS and to contribute to ER to mitochondria PS transfer for mitochondrial PS decarboxylation. This function of Mfn2 was necessary for ER phospholipid homeostasis, and liver Mfn2 ablation caused ER stress and inflammatory responses dependent on disruption of phospholipid biosynthesis. Considering these findings it may be suggested that ER-mitochondrial phospholipid and calcium transfer have opposing impacts during lipid overload-induced stress responses, explaining contradicting outcomes of manipulating ER-mitochondrial communication [61,62].

Macrophage lipid overload and an associated ER stress response are key pathologic features of atherosclerosis [66]. Advanced atherosclerotic plaques are characterized by necrotic cores, which mostly consist of dead, lipid-loaded macrophages. Cell death of macrophages by cholesterol overload involved the ER stress response, in particular its PERK-ATF4-CHOP branch [67]. One of the effectors of CHOP is ER oxidoreductin 1, with the further information that its increased expression resulted in oxidative hyper-activation of IP3R [68]. This in turn drove a cytosolic Ca^2+^ increase and activation of Ca^2+^/calmodulin-dependent protein kinase IIγ (CAMKIIγ), which promoted both extrinsic (Fas induction) and mitochondrial (cytochrome c release) branches of apoptosis [69]. In this model of ER-stress-triggered apoptosis mitochondrial depolarization and cytochrome c release were preceded by a mitochondrial Ca^2+^ overload. These alterations were also CAMKIIγ-dependent [69] and preventing mitochondrial Ca^2+^ uptake attenuated apoptosis. Furthermore, ER stress caused mitochondrial translocation of CAMKIIγ. Effects of disrupting ER-mitochondrial contacts were not specifically studied in this model and may result in ambivalent effects by either diverting IP3R-dependent Ca^2+^ flux to CAMKIIγ, or preventing mitochondrial Ca^2+^ overload. Since mROS may positively regulate CAMKIIγ through oxidation [69], attenuating mitochondrial Ca^2+^ accumulation and mROS generation may have a beneficial effect on the ER-stress-driven mitochondrial apoptotic pathways through reduced CAMKIIγ activity. 

**Figure 1:**
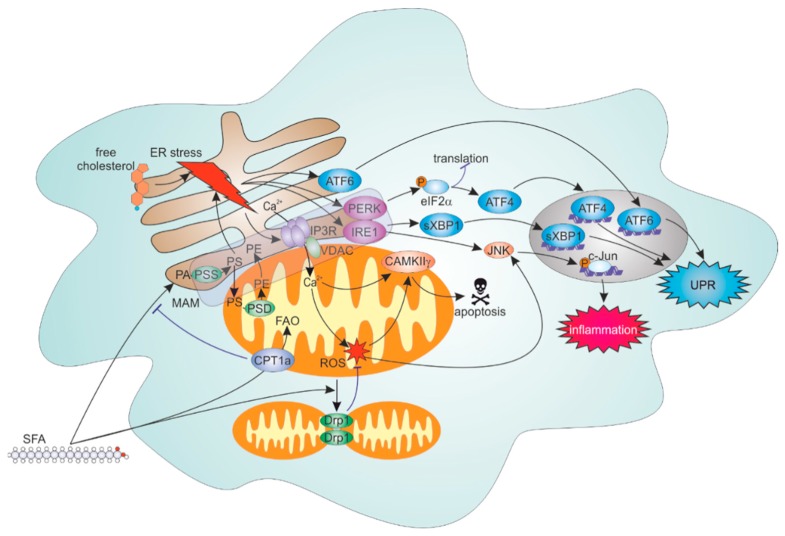
Endoplasmic reticulum (ER)-mitochondrial regulation of lipid overload-induced stress responses. Excessive incorporation of saturated fatty acids (SFA) into ER phospholipids or accumulation of free cholesterol in the ER induce ER stress response mediated by activating transcription factor 6 (ATF6), protein kinase RNA-activated (PKR)-like ER kinase (PERK) and inositol requiring enzyme 1 (IRE1) sensor proteins. Concomitantly, SFA undergo fatty acid β-oxidation (FAO), which attenuates SFA incorporation into phosphatidylserine (PS) and phosphatidylethanolamine (PE) and dampens ER stress. Furthermore, fatty acids induce Drp1-dependent mitochondrial fragmentation, which attenuates mitochondrial ROS formation and activation of pro-inflammatory c-Jun N-terminal kinase (JNK) signaling. ER stress also provokes inositol triphosphate receptors (IP3R) activation and mitochondrial Ca^2+^ overload. Under ER stress conditions, Ca^2+^/calmodulin-dependent protein kinase IIγ (CAMKIIγ) may translocate to mitochondria and undergo Ca^2+^- and ROS-dependent activation, promoting apoptosis. CPT: Carnitine palmitoyltransferase; PA: Phosphatidic acid; PSS: Phosphatidylserine synthase; PSD: Phosphatidylserine decarboxylase.

Collectively, accumulating literature suggests important contribution of ER, mitochondria, and their communication to shaping inflammatory and stress responses to lipid overload in macrophages. (mechanisms summarized in Figure 1). This adds to the growing evidence for disturbances of ER-mitochondrial contacts in the liver or skeletal muscle being crucial during development of insulin resistance and diabetes [61,62,70,71]. Still, there remains a substantial controversy towards the pathophysiological impact of tightening versus loosening of ER-mitochondrial interface, which needs to be resolved in future experiments [57]. Considering the relevance of ER-mitochondrial communication for the development of atherosclerosis, functional consequences of disrupting ER-mitochondrial contact sites on various aspects of the disease await evidence from in vivo models, such as tissue-specific knockouts of Mfn2.

## 5. ER-Mitochondria Contacts in Efferocytosis 

A prominent feature of macrophages is their ability to engulf and dispose of apoptotic cells (efferocytosis). Although most eukaryotic cells are engaged in efferocytosis, macrophages are particularly well suited for this process, able to rapidly engulf multiple apoptotic cells at once [72]. Efferocytosis allows an immunologically silent disposal of apoptotic cells, and macrophages dampen their inflammatory repertoire upon apoptotic cell clearance. Several recent studies documented metabolic rearrangements upon efferocytosis, which included alterations of mitochondrial metabolism. Thus, efferocytic macrophages increased fatty acid oxidation, which may facilitate anti-inflammatory cytokine, e.g. interleukin (IL)-10 production [73]. At the same time, efferocytosis increased aerobic glycolysis [74], promoting the uptake of multiple apoptotic bodies and fostering an anti-inflammatory environment through enhanced lactate release. A recent study [75] showed unexpected roles of mitochondrial dynamics and mitochondrial calcium in efferocytosis. Macrophages taking up apoptotic cells enhanced mitochondrial fragmentation, which was Drp1 dependent. Mitochondrial fragmentation prevented mitochondrial Ca^2+^ sequestration and thus, increased cytoplasmic Ca^2+^, which was necessary for efferocytic activity. Drp1 deficiency increased mitochondrial Ca^2+^, but decreased cytosolic Ca^2+^, thereby impairing efferocytosis. Silencing of MCU reversed these changes. Whether altering ER-mitochondria contacts and thus, Ca^2+^ transfer from the ER to mitochondria, affects efferocytosis remains unresolved. Efferocytosis is also dependent on mitochondrial membrane potential (ΔΨ_m_), with low ΔΨ_m_ induced by uncoupling protein 2 promoting apoptotic cell engulfment [76]. Exact mechanisms of this effect are unclear, and whether it relates to mitochondrial fragmentation needs to be proven. 

Recently, an interesting connection between lysosomal degradation of ingested material and mitochondrial lipid metabolism became apparent [77]. Inhibiting lysosomal acid lipase (LIPA) in macrophages engulfing apoptotic cells reduced oxidative phosphorylation, while increasing ΔΨ_m_ and generating mROS. LIPA inhibition caused NOD-like receptor protein 3 (NLRP3) inflammasome activation, which was sensitive to IP3R inhibition. Surprisingly, LIPA inhibition also decreased mitochondrial-ER contacts as assessed by transmission electron microscopy and diminished mitochondrial Ca^2+^. Overexpression of the ER chaperone Grp78 reversed the loss of mitochondrial Ca^2+^ and attenuated IL-1β secretion. Blocking LIPA also prevented formation of 25-hydroxycholesterol (25-OHC) in efferocytic macrophages. Addition of 25-OHC increased mitochondrial respiration, increased mitochondrial Ca^2+^, and prevented NLRP3 activation in LIPA-inhibitor-treated efferocytes, suggesting an unexpected link between ER cholesterol metabolism and mitochondrial activity in efferocytes. The mechanistic basis for this effect remains to be clarified. 

Impaired efferocytosis causes the development of autoimmune diseases, and is thought to contribute to necrotic core formation in advanced atherosclerotic lesions [78]. CAMKIIγ, in addition to promoting apoptosis of ER-stressed macrophages, negatively influenced efferocytosis [79]. CAMKIIγ-deficient macrophages expressed more liver X receptor α (LXRα) and its target Mer tyrosine kinase, increasing adherence and uptake of apoptotic cells. Interestingly, this increase was mediated by increased expression and activity of the UPR effector ATF6α in CAMKIIγ-deficient macrophages. Thus, under ER stress CAMKIIγ may shift the balance of UPR effector activation towards PERK and IRE1 branches, attenuating efferocytic capabilities of macrophages. 

## 6. ER-Mitochondria in Inflammasome

### 6.1. General Mechanism of NLRP3 Inflammasome Activation

Inflammasomes are multimolecular complexes, which transduce signals sensed by specific cytosolic proteins of the NLRP family into proteolytic activation of caspase-1 and -11. This results in cleavage and secretion of cytokines IL-1β, -1α, and -18 [80,81]. Also, caspase-1/-11-mediated cleavage of the cytosolic protein gasdermin D generates a *N*-terminal proteolytic fragment capable of creating pores in the plasma membrane thus, initiating pyroptotic cell death [82]. Among the inflammasome-activating sensors, NLRP3 is the most promiscuous, being activated under a variety of conditions, including disruption of ion fluxes, lysosomal membrane damage, and bacterial or viral infection. Exact mechanisms of NLRP3 activation are still not fully resolved, but generally involve a priming step through toll-like receptor (TLR) activation, increasing expression of pro-IL1β and NLRP3. Separate mechanisms in turn trigger assembly of multimeric complexes consisting of oligomers of NLRP3, adaptor protein apoptosis-associated speck-like protein containing a CARD (ASC), and caspase-1. Early on, ROS and K^+^ efflux were implicated in NLRP3 inflammasome activation [83,84]. The source of ROS activating NLRP3 was postulated to be mitochondria [85]. It is mechanistically still not exactly clear how mROS promotes NLRP3 activation, as their involvement is not universal to all NLRP3-activating stimuli [86]. mROS can contribute to inflammasome activation through oxidation and the cytosolic appearance of mtDNA [87]. ROS were also implicated in the inflammasome priming step [88] and IL-1β transcriptional activation [89]. On the other hand, K^+^ efflux is now accepted as the most general driver of inflammasome activation [80,86].

### 6.2. Mitochondria as Platforms for NLRP3 Inflammasome Assembly

In addition to their role in mROS generation, mitochondria are postulated as platforms for the recruitment of NLRP3 and subsequent inflammasome assembly. Cardiolipin, a major phospholipid of the mitochondrial inner membrane, directly bound NLRP3 and promoted caspase-1 activation when added to lipopolysaccharide (LPS)-primed, J774 macrophage lysates [90]. Interfering with cardiolipin biosynthesis attenuated NLRP3 mitochondrial translocation and IL-1β secretion in response to ATP or silica. How cardiolipin translocates from the inner to outer mitochondrial membrane (OMM) during inflammasome activation remained unclear. Further investigations showed that NLRP3 and caspase-1 mitochondrial translocation occurred during the priming step [91], whereas caspase-1 mitochondrial translocation happened independently of NLRP3, but also involved binding to cardiolipin at the OMM. Both NLRP3 mitochondrial recruitment and cardiolipin OMM translocation occurred in a ROS-dependent fashion. The activating step may then promote K^+^ efflux and Ca^2+^-dependent recruitment of ASC to mitochondrial NLRP3. 

A mitochondrial adaptor protein involved in innate immune responses to RNA viruses (see next section) mitochondrial antiviral-signaling protein (MAVS) mediated mitochondrial recruitment and activation of NLRP3 by canonical inflammasome stimuli, e.g. ATP and nigericin [92], or during viral infection [93,94,95]. Subsequent studies, however, failed to observe MAVS-dependency of NLRP3 activation by ATP or nigericin [93,96,97], restricting its role to viral infection-associated inflammasome activation. An indirect role of MAVS in NLRP3 inflammasome activation by viral dsRNA became apparent [96], thereby viral infection triggered MAVS-dependent plasma membrane permeabilization driving K^+^ efflux and NLRP3 activation.

### 6.3. ER-Mitochondrial Contacts Regulate NLRP3 Inflammasome

ER-mitochondrial contact sites were first suggested to provide platforms for NLRP3 inflammasome assembly in a study linking mROS to inflammasome activation [85]. Whereas NLRP3 showed cytosolic as well as partial ER localization in unstimulated THP-1 macrophages, it relocated together with ASC to MAM in response to the inflammasome activators nigericin, alum, or monosodium urate (MSU). Whether modulating the contacts between ER and mitochondria affects NLRP3 translocation and activation was, however, not tested in this study. 

Ca^2+^ transfer from the ER to mitochondria was suggested to contribute to inflammasome activation by stimuli such as ATP, nigericin, or MSU [98]. Inhibitors of phospholipase C, store-operated calcium entry, or IP3R prevented not only a cytosolic Ca^2+^ increase and IL-1β secretion, but also mROS generation, the loss of ΔΨ_m_, and mtDNA release into the cytosol, indicating that mitochondrial Ca^2+^ overload is a critical event to promote mitochondrial damage and mROS generation. Ca^2+^ entry was shown to be downstream of K^+^ efflux in ATP-stimulated cells. While this study did not specifically questioned whether mitochondrial Ca^2+^ entry is critical for the NLRP3 activation, MCU-mediated mitochondrial Ca^2+^ overload accompanied by mitochondrial NLRP3 translocation was shown to drive inflammasome activation by the complement membrane attack complex [99]. Whether this generally applies to canonical NLRP3-activating stimuli requires further investigation. At least in case of macrophages engulfing apoptotic cells NLRP3 activation was accompanied by decreased mitochondrial Ca^2+^, while being sensitive to IP3R inhibition, suggesting a cytosolic rather than mitochondrial Ca^2+^-involvement [77]. Whether these observations are specific to efferocytic macrophages remains to be established.

A study of NLRP3 inflammasome activation during RNA virus infection showed the importance of Mfn2 in this process [100]. Whereas RNA virus-triggered inflammasome did not require mROS, it was abolished in mtDNA-depleted macrophages, and attenuated by Mfn2 deficiency. Virus infection triggered the association of NLRP3 with Mfn1 and Mfn2 in LPS-primed BMDM in a ΔΨ_m_-dependent fashion. The authors proposed formation of a MAVS-Mfn2-NLRP3 ternary complex as a platform for inflammasome assembly. The role of Mfn2 as an ER-mitochondrial tether was not specifically addressed in this study, nor was the specificity of Mfn2 versus Mfn1 in inflammasome regulation investigated. Another mechanism how mitochondrial-ER contacts support inflammasome activation was suggested by Misawa et al. [101]. Inflammasome activators promoted the close interaction of mitochondrial ASC and ER-resident NLRP3 as proven by confocal microscopy and proximity ligation assays. Mechanistically, NLRP3 activators triggered tubulin acetylation, driven by deactivation of sirtuin 2 (SIRT2), a protein deacetylase, as a consequence of mitochondrial dysfunction and NAD^+^ loss. Acetylated tubulin promoted the dynein-driven approximation of ASC and NLRP3 in NLRP3-activator-treated macrophages. The locations of NLRP3 and ASC reported in this study for unstimulated macrophages unfortunately are not observed by others, which predominantly show cytosolic and nuclear ASC locations [81].

### 6.4. Mitophagy Dampens NLRP3 Inflammasome Activation

Safe elimination of damaged mitochondria is carried out by the organelle encirclement in autophagosomes, followed by their degradation in autolysosomes in a process known as mitophagy [102]. Several studies linked defective mitophagy to NLRP3 inflammasome activation in macrophages. Thus, SFA were reported to suppress the activity of mitophagy-activating AMP-activated protein kinase, which contributed to increased NLRP3 activity in cells exposed to SFA and LPS [103]. Attenuating mitophagy may promote NLRP3 activity through accumulation of mROS-producing mitochondria [85]. It was also reported to stimulate mROS- and NLRP3-dependent release of mtDNA, which then activated caspase-1 [104]. Furthermore, deletion of parkin, a ubiquitin E3 ligase central for mitophagy, increased NLRP3 inflammasome activity in macrophages and microglia cells [105,106]. Regarding the relation of ER-mitochondrial communication to mitophagy, conflicting data exist in the literature. ER-mitochondrial contact sites were suggested be the places of autophagosome formation during starvation-induced autophagy [32]. Although analysis of co-localization of fluorescent ER and mitochondrial markers revealed enhanced ER-mitochondrial contacts upon mitophagy-promoting stimuli [107], a more recent study using electron microscopy suggested the opposite effect [108]. Deleting or silencing ER-mitochondrial contact-promoting proteins Mfn2, phosphofurin acidic cluster sorting protein 2, or syntaxin 17, the authors showed attenuation of mitophagy driven by mitochondrial dysfunction in cells overexpressing parkin. ER-mitochondrial apposition was destroyed during mitophagy through parkin-dependent phosphoubiquitination of Mfn2 [108]. Conversely, enhanced ER-mitochondrial contacts were observed in parkin-deficient fibroblasts [109]. Whereas these data provided convincing evidence for the negative role of ER-mitochondrial tethering in the context of parkin-dependent mitophagy, it remains to be clarified whether disrupting ER-mitochondrial communication attenuates mitophagy under inflammasome-activating conditions in innate immune cells. This is particularly relevant for the nervous system, considering many links between ER-mitochondrial communication and neurodegenerative disease as well as neuroinflammatory disease [110]. 

**Figure 2:**
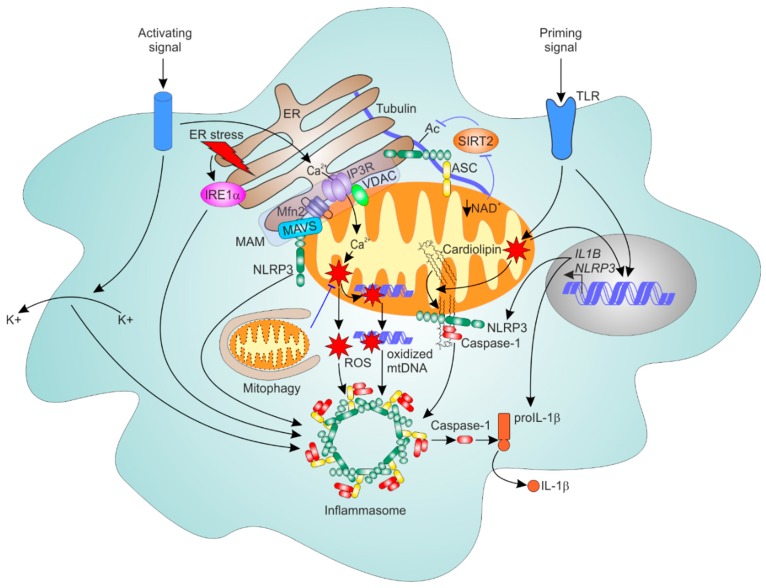
ER-mitochondrial communication in NOD-like receptor protein 3 (NLRP3) inflammasome regulation. NLRP3 inflammasome activation requires two signals; a priming signal (e.g. toll-like receptor (TLR) activation) induces the expression of pro-IL-1β and NLRP3, which occurs in a mROS-dependent manner. Priming signals also induce mROS-dependent cardiolipin translocation to the outer mitochondrial membrane (OMM), where it recruits NLRP3 and caspase-1. An activation signal via e.g. P2X7 receptor ligation promotes K^+^ efflux, mitochondrial Ca^2+^ increase, mROS elevation and release of oxidized mitochondrial DNA (mtDNA). These events contribute to the recruitment of ASC to NLRP3 and caspase-1 and formation of the activated oligomeric inflammasome complex, which cleaves and activates caspase-1, followed by the subsequent cleavage and secretion of interleukin (IL)-1β. Mitochondria-associated membranes (MAM) provide the platform for inflammasome assembly, which may be facilitated by the interaction of NLRP3, mitochondrial antiviral-signaling protein (MAVS) and mitofusin 2 (Mfn2). Movements of mitochondria and ER, driven by a loss of NAD^+^, SIRT2 inactivation, and tubulin acetylation may also facilitate apposition of NLRP3 and ASC. ER stress activates the NLRP3 inflammasome through IRE1α. Mitophagy may eliminate mROS-producing mitochondria and thus, dampen NLRP3 inflammasome activation.

As mentioned above, ER-mitochondrial communication may shape ER stress responses. ER stressors were shown to activate the NLRP3 inflammasome in THP-1 cells and primary human and murine macrophages. This required K^+^ efflux and ROS, but was independent of primary UPR effectors PERK, IRE1α, and ATF6 [111]. Whereas previous work suggested an indirect role of the UPR effector IRE1α in NLRP3 activation by ER stressors through microRNA miR-17 [112], more recent work suggested pathways linking ER stress and inflammasome to involve IRE1α-driven mitochondrial recruitment of NLRP3 [113]. The IRE1α branch of the ER stress response was also proposed to be involved in palmitate-induced NLRP3 inflammasome activation [114]. Whether ER-mitochondrial communication, in particular, ER-mitochondria Ca^2+^ transfer, influences ER stress-triggered NLRP3 inflammasome remains the topic of future investigation. We summarize the roles of ER and mitochondria in NLRP3 inflammasome regulation in Figure 2.

## 7. ER-Mitochondria Contacts and Antiviral Responses

### 7.1. Mechanisms of Cellular RNA Sensing 

Immune sensing of viral RNA and DNA is achieved by several TLR-proteins (TLR3, TLR7, TLR8, and TLR9), retinoic acid inducible gene I (RIG-I)-like receptor (RLR), and melanoma differentiation antigen 5 (MDA5) double-stranded RNA (dsRNA) sensor proteins, as well as by the cyclic GMP-AMP (cGAMP) synthase (cGAS)-stimulator of interferon genes (STING) pathway. Whereas TLR recognize nucleic acids in endolysosomes, RIG-I and MDA5 sense cytosolic dsRNA. Upon binding dsRNA, RIG-I, and MDA5 undergo conformational rearrangements, ubiquitination, and oligomerization, thereby allowing their interaction with the adaptor protein MAVS [115,116,117]. MAVS oligomerization and formation of prion-like structures [118] builds platforms for activation of interferon regulatory factor 3 (IRF3), IRF7, and NFκB transcription factors. MAVS localize to mitochondrial [119] as well as peroxisomal [120] membranes. Some viruses, such as hepatitis C virus (HCV), provoke MAVS mislocalization to attenuate antiviral signaling. Thus, the HCV protease NS3A-4A complex cleaves MAVS, releasing it into the cytosol and inhibiting RIG-I-mediated signaling [121,122].

### 7.2. ER and Mitochondria Control MAVS-Dependent RNA Sensing Mechanisms 

The role of ER-mitochondrial communication in RIG-MAVS sensing of RNA viruses was proposed in 2011 [123]. Using confocal microscopy and biochemical fractionation in HuH7 hepatoma cells, authors showed localization of MAVS in MAM together with typical MAM proteins such as acyl-CoA synthetase long chain family member 4, calnexin, or Mfn2. Interestingly, the authors suggested that MAM, but not mitochondria were sites of MAVS cleavage by NS3A-4A. Upon viral infection RIG-I accumulated in the MAM fraction. It was shown that Mfn2, but not Mfn1 knockdown, decreased co-localization of MAVS with mitochondria, while enhancing co-localization of MAVS with peroxisomes. Mfn2 silencing also enhanced virus-triggered phosphorylation of IRF3 and interferon target gene induction, whereas Mfn1 knockdown had an opposite effect. MAM targeting and MAVS interaction was recently suggested to be the mechanism how geranylgeranylated and palmitoylated GTPase Rac1 attenuates antiviral signaling [124]. Furthermore, an ER- and MAM-resident ubiquitin ligase gp78 was shown to interact with MAVS and promote its ubiquitination and degradation, attenuating antiviral responses [125]. A link between mitochondrial Ca^2+^ and RLR signaling was recently suggested, showing interactions of MAVS with MCU [126]. ER stress potentiated IRF3 phosphorylation and IFNβ induction in poly(I:C) treated HeLa cells in a manner dependent on MAVS and MCU as well as ROS.

The impact of mitofusins on MAVS-mediated viral sensing was addressed in the following studies. Overexpression of Mfn2 interfered with MAVS activation by viruses through locking MAVS in a non-productive state [127]. Conversely, Mfn2 silencing enhanced IFNβ production and IRF3 activation upon viral infection. Mfn2 was shown to directly interact with a C-terminal region of MAVS through its central hydrophobic region, suggesting that Mfn2-MAVS interaction is independent of Mfn2 functions in ER-mitochondrial tethering or mitochondrial fusion. Whereas this study found no evidence of Mfn1 involvement in MAVS signaling, others reported a role of mitochondrial dynamics in regulating RLR activity [128]. HeLa cells exposed to replication-deficient Sendai virus H4 strain or poly(I:C) showed mitochondrial elongation. Reducing mitochondrial fusion dampened activation of NFκB and IRF3 by the virus in luciferase reporter assays, whereas the opposite was observed after knocking down pro-fission proteins Drp1 and Fis1. This report also showed MAVS co-immunoprecipitation with Mfn1, but not with Mfn2, as well as alterations of ER morphology (“de-reticulation”) causing increased mitochondrial-ER co-localization upon viral infection. Yet another study suggested Mfn1 to support re-distribution of MAVS to the OMM to speckle-like aggregates, which may relate to the formation of prion-like structures [129]. On the other hand, a recent super-resolution microscopy study did not observe MAVS redistribution upon activation, whereas MAVS-dependent fragmentation and perinuclear accumulation of mitochondria was noticed [130]. It should be pointed out that the effect of viral infection on mitochondrial morphology may be dependent on replication competence [128]. MAVS signaling also requires intact ΔΨ_m_ [131] and it was suggested that this underlined the defect of antiviral signaling in MEFs deficient for both Mfn1 and Mfn2. The viruses dampened anti-viral responses through various mechanisms, some of them targeting MAM (reviewed in [132]).

In summary, depending on the type of the viral stimulus, the proteins mitofusins may impact RLR signaling both through their functions as ER-mitochondrial tethers as well as promotors of mitochondrial fusion. Further efforts are needed to clarify whether modulating the architecture of ER-mitochondrial contacts or altering ER to mitochondria Ca^2+^ and lipid transfer impacts viral RNA sensing. It should also be noted that most of the gathered information regarding roles of mitochondria and MAM in RLR signaling comes from data generated in non-phagocytic, mostly cancer cells. It remains to be clarified whether the mechanisms established for cancer cell lines are also operating in professional immune cells.

### 7.3. ER-Mitochondrial Regulation of Cytosolic DNA Sensing 

Innate immune sensing of cytosolic DNA operates predominantly through the cGAS-STING pathway [133]. STING is an ER-resident protein, which can be activated by bacterial cyclic dinucleotides or by cGAMP dinucleotide, a product of the enzyme cGAS. Upon cGAMP binding, STING undergoes conformational rearrangement, ubiquitination, binds to and gets phosphorylated by TANK-binding kinase 1 (TBK1). This complex translocates through the Golgi to perinuclear regions, where TBK1 activates IRF3 and NFκB pathways. In an initial report on STING, interactions of STING with MAVS and RIG-I were reported [134]. This interaction was documented in another early report [135]. However, this study suggested a mitochondrial location of STING, which was not confirmed later on. Subcellular fractionation showed STING to be partly present in the MAM fraction in unstimulated MEFs [136]. However, the importance of STING-MAVS interactions for antiviral responses is unclear. Recent findings suggest that STING may affect replication of RNA viruses independently of MAVS, whereas IFN-response to RNA viruses is unaffected by STING deficiency [137]. Whether the central mechanism of cGAMP sensing and response by STING is modulated by interactions with MAVS or ER-mitochondrial communication in general remains to be clarified. 

**Figure 3:**
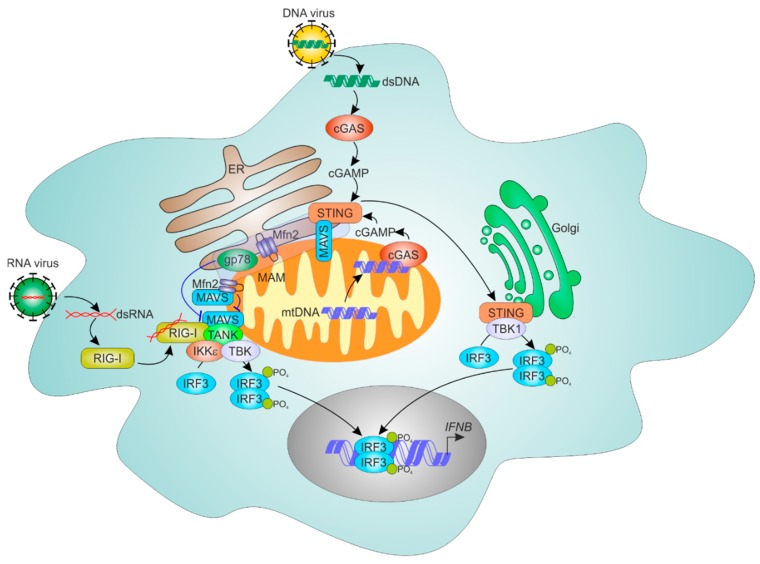
Communication of ER and mitochondrial in sensing cytosolic nucleic acids. The response to RNA viruses is orchestrated by the sensor retinoic acid inducible gene I (RIG-I), which upon dsRNA recognition translocates to mitochondria- and the MAM-located adaptor MAVS to activate the interferon regulatory factor 3 (IRF3) transcription factor. MAVS may be negatively regulated by their interaction with Mfn2 and Gp78. Sensing of cytosolic DNA involves activation of the cyclic GMP-AMP (cGAMP) synthase (cGAS)-stimulator of interferon genes (STING) cascade, whereas activated STING translocates from the ER to Golgi to activate IRF3. The cross-talk between DNA and RNA sensing may involve direct interaction of STING and MAVS. cGAS may also sense mtDNA following mitochondrial damage by translocating to mitochondria.

Apart from responding to bacterial or viral DNA, the cGAS-STING pathway senses nuclear and mitochondrial DNA damage [138]. Sensing mitochondrial damage by the cGAS-STING pathway involved cGAS translocation to mtDNA nucleoids, although mechanisms of translocation remains unclear [139]. The pathophysiological relevance of cGAS-STING sensing of endogenous mitochondrial damage and cytosolic mtDNA release is highlighted by observations of STING-dependent inflammation under stress conditions in mouse models of parkin and PTEN-induced kinase 1 deficiency, characterized by dysfunctional mitophagy [140]. The cell type primarily involved in sensing of mtDNA remains to be identified. We summarized roles of the ER and mitochondria in cellular responses to cytosolic nucleic acids in Figure 3. 

## 8. Concluding Remarks 

Recent years considerably advanced our understanding of molecular events governing crucial immune reactions, such as antiviral responses or bacterial pathogen recognition. Mitochondria and the ER make significant contribution to anti-viral and anti-bacterial signaling. Although we appreciate the importance of the ER-mitochondrial communication for cellular metabolism and signaling, its specific contribution to immune responses was rarely addressed. This is obvious considering the paucity of the studies directly addressing the impact of disturbed ER-mitochondrial tethering on processes affecting the innate immune system. Most information so far comes from studies using non-classical immune cells (Table 1). Still, recent data gathered in macrophages highlight phenomena with potential contributions of ER-mitochondrial tethering (Table 2), which need to be addressed in future studies. Furthermore, with an ever-growing list of proteins mediating tethering of ER to mitochondria and cargo transfer between these two organelles, the specific functions of these proteins in shaping innate immune responses remain to be elucidated. For example, the contribution of ER-mitochondria communication to efferocytosis or antigen presentation still has to be addressed. Last, we know very little about the roles of ER and mitochondria in neutrophils or in less abundant innate immune cell types, such as mast cells, eosinophils, or natural killer cells. The significance of ER-mitochondrial contacts will surely expand and embrace many new immunological aspects in the future. 

## Figures and Tables

**Table 1:** Effects of modulating ER-mitochondrial tethering on ER stress and antiviral responses.

Modulation	Effect on Tethering	Physiological Outcome
**ER Stress Responses**		
silencing Grp75, Mfn2, inhibiting cyclophilin D	decrease	increased ER stress and reduced insulin signaling in the liver [61]
overexpression of ER-mitochondrial tether in the liver	increase	mitochondrial Ca^2+^ overload, ROS formation, reduced insulin signaling [62]
liver-specific Mfn2 knockout	decrease	Disturbed ER phospholipid homeostasis, ER stress, inflammation [65]
**Antiviral Response**		
Mfn2 silencing	decrease	increased antiviral signaling in hepatoma and HEK 293 cells [123,127]

**Table 2:** Potential impact of ER-mitochondrial tethering in macrophages.

Modulation	Potential Impact of Increased Tethering	Physiological Outcome
**ER Stress Responses**		
FAO	increase	attenuated ER stress and inflammation induced by SFA [50]
mitochondrial fragmentation induced by fatty acids	increase	attenuated mROS formation and inflammation in response to SFA [52]
mitochondrial CAMKIIγ translocation following ER stress	increase	mitochondrial Ca^2+^ overload, ΔΨ_m_ loss, apoptosis [69]
**Efferocytosis**		
mitochondrial fragmentation following efferocytosis	increase	reduced mitochondrial Ca^2+^ uptake, enhanced efferocytosis [75]
LIPA- and 25-hydroxycholesterol-driven mitochondrial respiration	increase	attenuated mROS, increased mitochondrial Ca^2+^ and ΔΨ_m_, attenuated NLRP3 activity [77]
**Inflammasome Activation**		
ER to mitochondria Ca^2+^ transfer	increase	NLRP3 activation [98,99]
mROS generation	increase	NLRP3 activation [85]
cardiolipin OMM translocation	unclear	NLRP3 activation [90,91]
mitophagy	decrease	NLRP3 inhibition [85,103,104,105]

## References

[1] Gatta A.T., Levine T.P. (2017). Piecing together the patchwork of contact sites. Trends Cell Biol..

[2] Prinz W.A. (2014). Bridging the gap: Membrane contact sites in signaling, metabolism, and organelle dynamics. J. Cell Biol..

[3] Vance J.E. (1990). Phospholipid synthesis in a membrane fraction associated with mitochondria. J. Biol. Chem..

[4] Rusiñol A.E., Cui Z., Chen M.H., Vance J.E. (1994). A unique mitochondria-associated membrane fraction from rat liver has a high capacity for lipid synthesis and contains pre-Golgi secretory proteins including nascent lipoproteins. J. Biol. Chem..

[5] Lackner L.L. (2019). The expanding and unexpected functions of mitochondria contact sites. Trends Cell Biol..

[6] Csordás G., Weaver D., Hajnóczky G. (2018). Endoplasmic reticulum-mitochondrial contactology: Structure and Signaling Functions. Trends Cell Biol..

[7] Kornmann B., Currie E., Collins S.R., Schuldiner M., Nunnari J., Weissman J.S., Walter P. (2009). An ER-mitochondria tethering complex revealed by a synthetic biology screen. Science.

[8] Hirabayashi Y., Kwon S.-K., Paek H., Pernice W.M., Paul M.A., Lee J., Erfani P., Raczkowski A., Petrey D.S., Pon L.A. (2017). ER-mitochondria tethering by PDZD8 regulates Ca^2+^ dynamics in mammalian neurons. Science.

[9] de Brito O.M., Scorrano L. (2008). Mitofusin 2 tethers endoplasmic reticulum to mitochondria. Nature.

[10] Filadi R., Greotti E., Turacchio G., Luini A., Pozzan T., Pizzo P. (2015). Mitofusin 2 ablation increases endoplasmic reticulum-mitochondria coupling. Proc. Natl. Acad. Sci. USA.

[11] Naon D., Zaninello M., Giacomello M., Varanita T., Grespi F., Lakshminaranayan S., Serafini A., Semenzato M., Herkenne S., Hernández-Alvarez M.I. (2016). Critical reappraisal confirms that Mitofusin 2 is an endoplasmic reticulum-mitochondria tether. Proc. Natl. Acad. Sci. USA.

[12] Schrepfer E., Scorrano L. (2016). Mitofusins, from Mitochondria to metabolism. Mol. Cell.

[13] Marchi S., Patergnani S., Missiroli S., Morciano G., Rimessi A., Wieckowski M.R., Giorgi C., Pinton P. (2018). Mitochondrial and endoplasmic reticulum calcium homeostasis and cell death. Cell Calcium.

[14] Patergnani S., Suski J.M., Agnoletto C., Bononi A., Bonora M., de Marchi E., Giorgi C., Marchi S., Missiroli S., Poletti F. (2011). Calcium signaling around mitochondria associated membranes (MAMs). Cell Commun. Signal..

[15] Rizzuto R., Brini M., Murgia M., Pozzan T. (1993). Microdomains with high Ca2+ close to IP3-sensitive channels that are sensed by neighboring mitochondria. Science.

[16] Csordás G., Thomas A.P., Hajnóczky G. (1999). Quasi-synaptic calcium signal transmission between endoplasmic reticulum and mitochondria. EMBO J..

[17] Szabadkai G., Bianchi K., Várnai P., de Stefani D., Wieckowski M.R., Cavagna D., Nagy A.I., Balla T., Rizzuto R. (2006). Chaperone-mediated coupling of endoplasmic reticulum and mitochondrial Ca2+ channels. J. Cell Biol..

[18] Hayashi T., Su T.-P. (2007). Sigma-1 receptor chaperones at the ER-mitochondrion interface regulate Ca(^2+^) signaling and cell survival. Cell.

[19] Kuo I.Y., Brill A.L., Lemos F.O., Jiang J.Y., Falcone J.L., Kimmerling E.P., Cai Y., Dong K., Kaplan D.L., Wallace D.P. (2019). Polycystin 2 regulates mitochondrial Ca^2+^ signaling, bioenergetics, and dynamics through mitofusin 2. Sci. Signal..

[20] Phillips M.J., Voeltz G.K. (2016). Structure and function of ER membrane contact sites with other organelles. Nat. Rev. Mol. Cell Biol..

[21] Friedman J.R., Lackner L.L., West M., DiBenedetto J.R., Nunnari J., Voeltz G.K. (2011). ER tubules mark sites of mitochondrial division. Science.

[22] Lewis S.C., Uchiyama L.F., Nunnari J. (2016). ER-mitochondria contacts couple mtDNA synthesis with mitochondrial division in human cells. Science.

[23] Vance J.E. (2014). MAM (mitochondria-associated membranes) in mammalian cells: Lipids and beyond. Biochim. Biophys. Acta.

[24] Vance J.E. (1991). Newly made phosphatidylserine and phosphatidylethanolamine are preferentially translocated between rat liver mitochondria and endoplasmic reticulum. J. Biol. Chem..

[25] Shiao Y.J., Lupo G., Vance J.E. (1995). Evidence that phosphatidylserine is imported into mitochondria via a mitochondria-associated membrane and that the majority of mitochondrial phosphatidylethanolamine is derived from decarboxylation of phosphatidylserine. J. Biol. Chem..

[26] Lahiri S., Chao J.T., Tavassoli S., Wong A.K., Choudhary V., Young B.P., Loewen C.J., Prinz W.A. (2014). A conserved endoplasmic reticulum membrane protein complex (EMC) facilitates phospholipid transfer from the ER to mitochondria. PLoS Biol..

[27] Galmes R., Houcine A., van Vliet A.R., Agostinis P., Jackson C.L., Giordano F. (2016). ORP5/ORP8 localize to endoplasmic reticulum-mitochondria contacts and are involved in mitochondrial function. EMBO Rep..

[28] Kumar N., Leonzino M., Hancock-Cerutti W., Horenkamp F.A., Li P., Lees J.A., Wheeler H., Reinisch K.M., de Camilli P. (2018). VPS13A and VPS13C are lipid transport proteins differentially localized at ER contact sites. J. Cell Biol..

[29] Giordano F. (2018). Non-vesicular lipid trafficking at the endoplasmic reticulum-mitochondria interface. Biochem. Soc. Trans..

[30] Prudent J., McBride H.M. (2017). The mitochondria-endoplasmic reticulum contact sites: A signalling platform for cell death. Curr. Opin. Cell Biol..

[31] Prudent J., Zunino R., Sugiura A., Mattie S., Shore G.C., McBride H.M. (2015). MAPL SUMOylation of Drp1 Stabilizes an ER/Mitochondrial Platform Required for Cell Death. Mol. Cell.

[32] Hamasaki M., Furuta N., Matsuda A., Nezu A., Yamamoto A., Fujita N., Oomori H., Noda T., Haraguchi T., Hiraoka Y. (2013). Autophagosomes form at ER-mitochondria contact sites. Nature.

[33] Betz C., Stracka D., Prescianotto-Baschong C., Frieden M., Demaurex N., Hall M.N. (2013). Feature Article: mTOR complex 2-Akt signaling at mitochondria-associated endoplasmic reticulum membranes (MAM) regulates mitochondrial physiology. Proc. Natl. Acad. Sci. USA.

[34] van den Bossche J., O’Neill L.A., Menon D. (2017). Macrophage immunometabolism: Where are we (Going)?. Trends Immunol..

[35] Remmerie A., Scott C.L. (2018). Macrophages and lipid metabolism. Cell. Immunol..

[36] Tabas I., Bornfeldt K.E. (2016). Macrophage Phenotype and function in different stages of atherosclerosis. Circ. Res..

[37] Namgaladze D., Brüne B. (2016). Macrophage fatty acid oxidation and its roles in macrophage polarization and fatty acid-induced inflammation. Biochim. Biophys. Acta.

[38] Norris P.C., Dennis E.A. (2014). A lipidomic perspective on inflammatory macrophage eicosanoid signaling. Adv. Biol. Regul..

[39] Buckley C.D., Gilroy D.W., Serhan C.N. (2014). Proresolving lipid mediators and mechanisms in the resolution of acute inflammation. Immunity.

[40] Hetz C., Papa F.R. (2018). The unfolded protein response and cell fate control. Mol. Cell.

[41] Volmer R., van der Ploeg K., Ron D. (2013). Membrane lipid saturation activates endoplasmic reticulum unfolded protein response transducers through their transmembrane domains. Proc. Natl. Acad. Sci. USA.

[42] Li Y., Ge M., Ciani L., Kuriakose G., Westover E.J., Dura M., Covey D.F., Freed J.H., Maxfield F.R., Lytton J. (2004). Enrichment of endoplasmic reticulum with cholesterol inhibits sarcoplasmic-endoplasmic reticulum calcium ATPase-2b activity in parallel with increased order of membrane lipids: Implications for depletion of endoplasmic reticulum calcium stores and apoptosis in cholesterol-loaded macrophages. J. Biol. Chem..

[43] Håversen L., Danielsson K.N., Fogelstrand L., Wiklund O. (2009). Induction of proinflammatory cytokines by long-chain saturated fatty acids in human macrophages. Atherosclerosis.

[44] Erbay E., Babaev V.R., Mayers J.R., Makowski L., Charles K.N., Snitow M.E., Fazio S., Wiest M.M., Watkins S.M., Linton M.F. (2009). Reducing endoplasmic reticulum stress through a macrophage lipid chaperone alleviates atherosclerosis. Nat. Med..

[45] Palomer X., Pizarro-Delgado J., Barroso E., Vázquez-Carrera M. (2018). Palmitic and oleic acid: The yin and yang of fatty acids in type 2 diabetes mellitus. Trends Endocrinol. Metab..

[46] Solinas G., Naugler W., Galimi F., Lee M.-S., Karin M. (2006). Saturated fatty acids inhibit induction of insulin gene transcription by JNK-mediated phosphorylation of insulin-receptor substrates. Proc. Natl. Acad. Sci. USA.

[47] Lee J.Y., Sohn K.H., Rhee S.H., Hwang D. (2001). Saturated fatty acids, but not unsaturated fatty acids, induce the expression of cyclooxygenase-2 mediated through Toll-like receptor 4. J. Biol. Chem..

[48] Urano F., Wang X., Bertolotti A., Zhang Y., Chung P., Harding H.P., Ron D. (2000). Coupling of stress in the ER to activation of JNK protein kinases by transmembrane protein kinase IRE1. Science.

[49] Jiang H.-Y., Wek S.A., McGrath B.C., Scheuner D., Kaufman R.J., Cavener D.R., Wek R.C. (2003). Phosphorylation of the alpha subunit of eukaryotic initiation factor 2 is required for activation of NF-kappaB in response to diverse cellular stresses. Mol. Cell. Biol..

[50] Namgaladze D., Lips S., Leiker T.J., Murphy R.C., Ekroos K., Ferreiros N., Geisslinger G., Brüne B. (2014). Inhibition of macrophage fatty acid β-oxidation exacerbates palmitate-induced inflammatory and endoplasmic reticulum stress responses. Diabetologia.

[51] Gonzalez-Hurtado E., Lee J., Choi J., Selen Alpergin E.S., Collins S.L., Horton M.R., Wolfgang M.J. (2017). Loss of macrophage fatty acid oxidation does not potentiate systemic metabolic dysfunction. Am. J. Physiol. Endocrinol. Metab..

[52] Zezina E., Snodgrass R.G., Schreiber Y., Zukunft S., Schürmann C., Heringdorf D.M.Z., Geisslinger G., Fleming I., Brandes R.P., Brüne B. (2018). Mitochondrial fragmentation in human macrophages attenuates palmitate-induced inflammatory responses. Biochim. Biophys. Acta Mol. Cell Biol. Lipids.

[53] Snodgrass R.G., Boß M., Zezina E., Weigert A., Dehne N., Fleming I., Brüne B., Namgaladze D. (2016). Hypoxia potentiates palmitate-induced pro-inflammatory activation of primary human macrophages. J. Biol. Chem..

[54] Holzer R.G., Park E.-J., Li N., Tran H., Chen M., Choi C., Solinas G., Karin M. (2011). Saturated fatty acids induce c-Src clustering within membrane subdomains, leading to JNK activation. Cell.

[55] Kant S., Standen C.L., Morel C., Jung D.Y., Kim J.K., Swat W., Flavell R.A., Davis R.J. (2017). A Protein scaffold coordinates SRC-mediated JNK Activation in response to metabolic stress. Cell Rep..

[56] Garaude J., Acín-Pérez R., Martínez-Cano S., Enamorado M., Ugolini M., Nistal-Villán E., Hervás-Stubbs S., Pelegrín P., Sander L.E., Enríquez J.A. (2016). Mitochondrial respiratory-chain adaptations in macrophages contribute to antibacterial host defense. Nat. Immunol..

[57] Tubbs E., Rieusset J. (2017). Metabolic signaling functions of ER-mitochondria contact sites: Role in metabolic diseases. J. Mol. Endocrinol..

[58] Mori T., Hayashi T., Hayashi E., Su T.-P. (2013). Sigma-1 receptor chaperone at the ER-mitochondrion interface mediates the mitochondrion-ER-nucleus signaling for cellular survival. PLoS ONE.

[59] Verfaillie T., Rubio N., Garg A.D., Bultynck G., Rizzuto R., Decuypere J.-P., Piette J., Linehan C., Gupta S., Samali A. (2012). PERK is required at the ER-mitochondrial contact sites to convey apoptosis after ROS-based ER stress. Cell Death Differ..

[60] Carreras-Sureda A., Jaña F., Urra H., Durand S., Mortenson D.E., Sagredo A., Bustos G., Hazari Y., Ramos-Fernández E., Sassano M.L. (2019). Non-canonical function of IRE1α determines mitochondria-associated endoplasmic reticulum composition to control calcium transfer and bioenergetics. Nat. Cell Biol..

[61] Tubbs E., Theurey P., Vial G., Bendridi N., Bravard A., Chauvin M.-A., Ji-Cao J., Zoulim F., Bartosch B., Ovize M. (2014). Mitochondria-associated endoplasmic reticulum membrane (MAM) integrity is required for insulin signaling and is implicated in hepatic insulin resistance. Diabetes.

[62] Arruda A.P., Pers B.M., Parlakgül G., Güney E., Inouye K., Hotamisligil G.S. (2014). Chronic enrichment of hepatic endoplasmic reticulum-mitochondria contact leads to mitochondrial dysfunction in obesity. Nat. Med..

[63] Sebastián D., Hernández-Alvarez M.I., Segalés J., Sorianello E., Muñoz J.P., Sala D., Waget A., Liesa M., Paz J.C., Gopalacharyulu P. (2012). Mitofusin 2 (Mfn2) links mitochondrial and endoplasmic reticulum function with insulin signaling and is essential for normal glucose homeostasis. Proc. Natl. Acad. Sci. USA.

[64] Muñoz J.P., Ivanova S., Sánchez-Wandelmer J., Martínez-Cristóbal P., Noguera E., Sancho A., Díaz-Ramos A., Hernández-Alvarez M.I., Sebastián D., Mauvezin C. (2013). Mfn2 modulates the UPR and mitochondrial function via repression of PERK. EMBO J..

[65] Hernández-Alvarez M.I., Sebastián D., Vives S., Ivanova S., Bartoccioni P., Kakimoto P., Plana N., Veiga S.R., Hernández V., Vasconcelos N. (2019). Deficient Endoplasmic reticulum-mitochondrial phosphatidylserine transfer causes liver disease. Cell.

[66] Tabas I. (2010). The role of endoplasmic reticulum stress in the progression of atherosclerosis. Circ. Res..

[67] Feng B., Yao P.M., Li Y., Devlin C.M., Zhang D., Harding H.P., Sweeney M., Rong J.X., Kuriakose G., Fisher E.A. (2003). The endoplasmic reticulum is the site of cholesterol-induced cytotoxicity in macrophages. Nat. Cell Biol..

[68] Li G., Mongillo M., Chin K.-T., Harding H., Ron D., Marks A.R., Tabas I. (2009). Role of ERO1-alpha-mediated stimulation of inositol 1,4,5-triphosphate receptor activity in endoplasmic reticulum stress-induced apoptosis. J. Cell Biol..

[69] Timmins J.M., Ozcan L., Seimon T.A., Li G., Malagelada C., Backs J., Backs T., Bassel-Duby R., Olson E.N., Anderson M.E. (2009). Calcium/calmodulin-dependent protein kinase II links ER stress with Fas and mitochondrial apoptosis pathways. J. Clin. Invest..

[70] Tubbs E., Chanon S., Robert M., Bendridi N., Bidaux G., Chauvin M.-A., Ji-Cao J., Durand C., Gauvrit-Ramette D., Vidal H. (2018). Disruption of mitochondria-associated endoplasmic reticulum membrane (MAM) Integrity contributes to muscle insulin resistance in mice and humans. Diabetes.

[71] Thoudam T., Ha C.-M., Leem J., Chanda D., Park J.-S., Kim H.-J., Jeon J.-H., Choi Y.-K., Liangpunsakul S., Huh Y.H. (2019). PDK4 Augments ER-mitochondria contact to dampen skeletal muscle insulin signaling during obesity. Diabetes.

[72] Morioka S., Maueröder C., Ravichandran K.S. (2019). Living on the edge: efferocytosis at the interface of homeostasis and pathology. Immunity.

[73] Zhang S., Weinberg S., DeBerge M., Gainullina A., Schipma M., Kinchen J.M., Ben-Sahra I., Gius D.R., Yvan-Charvet L., Chandel N.S. (2019). Efferocytosis fuels requirements of fatty acid oxidation and the electron transport chain to polarize macrophages for tissue repair. Cell Metab..

[74] Morioka S., Perry J.S.A., Raymond M.H., Medina C.B., Zhu Y., Zhao L., Serbulea V., Onengut-Gumuscu S., Leitinger N., Kucenas S. (2018). Efferocytosis induces a novel SLC program to promote glucose uptake and lactate release. Nature.

[75] Wang Y., Subramanian M., Yurdagul A., Barbosa-Lorenzi V.C., Cai B., de Juan-Sanz J., Ryan T.A., Nomura M., Maxfield F.R., Tabas I. (2017). Mitochondrial fission promotes the continued clearance of apoptotic cells by macrophages. Cell.

[76] Park D., Han C.Z., Elliott M.R., Kinchen J.M., Trampont P.C., Das S., Collins S., Lysiak J.J., Hoehn K.L., Ravichandran K.S. (2011). Continued clearance of apoptotic cells critically depends on the phagocyte Ucp2 protein. Nature.

[77] Viaud M., Ivanov S., Vujic N., Duta-Mare M., Aira L.-E., Barouillet T., Garcia E., Orange F., Dugail I., Hainault I. (2018). Lysosomal cholesterol hydrolysis couples efferocytosis to anti-inflammatory oxysterol production. Circ. Res..

[78] Tabas I. (2010). Macrophage death and defective inflammation resolution in atherosclerosis. Nat. Rev. Immunol..

[79] Doran A.C., Ozcan L., Cai B., Zheng Z., Fredman G., Rymond C.C., Dorweiler B., Sluimer J.C., Hsieh J., Kuriakose G. (2017). CAMKIIγ suppresses an efferocytosis pathway in macrophages and promotes atherosclerotic plaque necrosis. J. Clin. Invest..

[80] He Y., Hara H., Núñez G. (2016). Mechanism and regulation of NLRP3 Inflammasome activation. Trends Biochem. Sci..

[81] Yabal M., Calleja D.J., Simpson D.S., Lawlor K.E. (2019). Stressing out the mitochondria: Mechanistic insights into NLRP3 inflammasome activation. J. Leukoc. Biol..

[82] Shi J., Gao W., Shao F. (2017). Pyroptosis: Gasdermin-mediated programmed necrotic cell death. Trends Biochem. Sci..

[83] Cruz C.M., Rinna A., Forman H.J., Ventura A.L.M., Persechini P.M., Ojcius D.M. (2007). ATP activates a reactive oxygen species-dependent oxidative stress response and secretion of proinflammatory cytokines in macrophages. J. Biol. Chem..

[84] Pétrilli V., Papin S., Dostert C., Mayor A., Martinon F., Tschopp J. (2007). Activation of the NALP3 inflammasome is triggered by low intracellular potassium concentration. Cell Death Differ..

[85] Zhou R., Yazdi A.S., Menu P., Tschopp J. (2011). A role for mitochondria in NLRP3 inflammasome activation. Nature.

[86] Muñoz-Planillo R., Kuffa P., Martínez-Colón G., Smith B.L., Rajendiran T.M., Núñez G. (2013). K⁺ efflux is the common trigger of NLRP3 inflammasome activation by bacterial toxins and particulate matter. Immunity.

[87] Shimada K., Crother T.R., Karlin J., Dagvadorj J., Chiba N., Chen S., Ramanujan V.K., Wolf A.J., Vergnes L., Ojcius D.M. (2012). Oxidized mitochondrial DNA activates the NLRP3 inflammasome during apoptosis. Immunity.

[88] Bauernfeind F., Bartok E., Rieger A., Franchi L., Núñez G., Hornung V. (2011). Cutting edge: Reactive oxygen species inhibitors block priming, but not activation, of the NLRP3 inflammasome. J. Immunol..

[89] Mills E.L., Kelly B., Logan A., Costa A.S.H., Varma M., Bryant C.E., Tourlomousis P., Däbritz J.H.M., Gottlieb E., Latorre I. (2016). Succinate dehydrogenase supports metabolic repurposing of mitochondria to drive inflammatory macrophages. Cell.

[90] Iyer S.S., He Q., Janczy J.R., Elliott E.I., Zhong Z., Olivier A.K., Sadler J.J., Knepper-Adrian V., Han R., Qiao L. (2013). Mitochondrial cardiolipin is required for Nlrp3 inflammasome activation. Immunity.

[91] Elliott E.I., Miller A.N., Banoth B., Iyer S.S., Stotland A., Weiss J.P., Gottlieb R.A., Sutterwala F.S., Cassel S.L. (2018). Cutting edge: Mitochondrial assembly of the NLRP3 inflammasome complex is initiated at priming. J. Immunol..

[92] Subramanian N., Natarajan K., Clatworthy M.R., Wang Z., Germain R.N. (2013). The adaptor MAVS promotes NLRP3 mitochondrial localization and inflammasome activation. Cell.

[93] Park S., Juliana C., Hong S., Datta P., Hwang I., Fernandes-Alnemri T., Yu J.-W., Alnemri E.S. (2013). The mitochondrial antiviral protein MAVS associates with NLRP3 and regulates its inflammasome activity. J. Immunol..

[94] Ermler M.E., Traylor Z., Patel K., Schattgen S.A., Vanaja S.K., Fitzgerald K.A., Hise A.G. (2014). Rift Valley fever virus infection induces activation of the NLRP3 inflammasome. Virology.

[95] Chakrabarti A., Banerjee S., Franchi L., Loo Y.-M., Gale M., Núñez G., Silverman R.H. (2015). RNase L activates the NLRP3 inflammasome during viral infections. Cell Host Microbe.

[96] Franchi L., Eigenbrod T., Muñoz-Planillo R., Ozkurede U., Kim Y.-G., Arindam C., Gale M., Silverman R.H., Colonna M., Akira S. (2014). Cytosolic double-stranded RNA activates the NLRP3 inflammasome via MAVS-induced membrane permeabilization and K+ efflux. J. Immunol..

[97] Allam R., Lawlor K.E., Yu E.C.-W., Mildenhall A.L., Moujalled D.M., Lewis R.S., Ke F., Mason K.D., White M.J., Stacey K.J. (2014). Mitochondrial apoptosis is dispensable for NLRP3 inflammasome activation but non-apoptotic caspase-8 is required for inflammasome priming. EMBO Rep..

[98] Murakami T., Ockinger J., Yu J., Byles V., McColl A., Hofer A.M., Horng T. (2012). Critical role for calcium mobilization in activation of the NLRP3 inflammasome. Proc. Natl. Acad. Sci. USA..

[99] Triantafilou K., Hughes T.R., Triantafilou M., Morgan B.P. (2013). The complement membrane attack complex triggers intracellular Ca2+ fluxes leading to NLRP3 inflammasome activation. J. Cell Sci..

[100] Ichinohe T., Yamazaki T., Koshiba T., Yanagi Y. (2013). Mitochondrial protein mitofusin 2 is required for NLRP3 inflammasome activation after RNA virus infection. Proc. Natl. Acad. Sci. USA.

[101] Misawa T., Takahama M., Kozaki T., Lee H., Zou J., Saitoh T., Akira S. (2013). Microtubule-driven spatial arrangement of mitochondria promotes activation of the NLRP3 inflammasome. Nat. Immunol..

[102] Pickles S., Vigié P., Youle R.J. (2018). Mitophagy and quality control mechanisms in mitochondrial maintenance. Current Biology.

[103] Wen H., Gris D., Lei Y., Jha S., Zhang L., Huang M.T.-H., Brickey W.J., Ting J.P.-Y. (2011). Fatty acid-induced NLRP3-ASC inflammasome activation interferes with insulin signaling. Nat. Immunol..

[104] Nakahira K., Haspel J.A., Rathinam V.A.K., Lee S.-J., Dolinay T., Lam H.C., Englert J.A., Rabinovitch M., Cernadas M., Kim H.P. (2011). Autophagy proteins regulate innate immune responses by inhibiting the release of mitochondrial DNA mediated by the NALP3 inflammasome. Nat. Immunol..

[105] Zhong Z., Umemura A., Sanchez-Lopez E., Liang S., Shalapour S., Wong J., He F., Boassa D., Perkins G., Ali S.R. (2016). NF-κB Restricts Inflammasome Activation via Elimination of Damaged Mitochondria. Cell.

[106] Mouton-Liger F., Rosazza T., Sepulveda-Diaz J., Ieang A., Hassoun S.-M., Claire E., Mangone G., Brice A., Michel P.P., Corvol J.-C. (2018). Parkin deficiency modulates NLRP3 inflammasome activation by attenuating an A20-dependent negative feedback loop. Glia.

[107] Gelmetti V., de Rosa P., Torosantucci L., Marini E.S., Romagnoli A., Di Rienzo M., Arena G., Vignone D., Fimia G.M., Valente E.M. (2017). PINK1 and BECN1 relocalize at mitochondria-associated membranes during mitophagy and promote ER-mitochondria tethering and autophagosome formation. Autophagy.

[108] McLelland G.-L., Goiran T., Yi W., Dorval G., Chen C.X., Lauinger N.D., Krahn A.I., Valimehr S., Rakovic A., Rouiller I. (2018). Mfn2 ubiquitination by PINK1/parkin gates the p97-dependent release of ER from mitochondria to drive mitophagy. Elife.

[109] Gautier C.A., Erpapazoglou Z., Mouton-Liger F., Muriel M.P., Cormier F., Bigou S., Duffaure S., Girard M., Foret B., Iannielli A. (2016). The endoplasmic reticulum-mitochondria interface is perturbed in PARK2 knockout mice and patients with PARK2 mutations. Hum. Mol. Genet..

[110] Erpapazoglou Z., Mouton-Liger F., Corti O. (2017). From dysfunctional endoplasmic reticulum-mitochondria coupling to neurodegeneration. Neurochemistry International.

[111] Menu P., Mayor A., Zhou R., Tardivel A., Ichijo H., Mori K., Tschopp J. (2012). ER stress activates the NLRP3 inflammasome via an UPR-independent pathway. Cell Death Dis..

[112] Lerner A.G., Upton J.-P., Praveen P.V.K., Ghosh R., Nakagawa Y., Igbaria A., Shen S., Nguyen V., Backes B.J., Heiman M. (2012). IRE1α induces thioredoxin-interacting protein to activate the NLRP3 inflammasome and promote programmed cell death under irremediable ER stress. Cell Metab..

[113] Bronner D.N., Abuaita B.H., Chen X., Fitzgerald K.A., Nuñez G., He Y., Yin X.-M., O’Riordan M.X.D. (2015). Endoplasmic reticulum stress activates the inflammasome via NLRP3- and Caspase-2-driven mitochondrial damage. Immunity.

[114] Robblee M.M., Kim C.C., Porter Abate J., Valdearcos M., Sandlund K.L.M., Shenoy M.K., Volmer R., Iwawaki T., Koliwad S.K. (2016). Saturated fatty acids engage an IRE1α-dependent pathway to activate the NLRP3 inflammasome in myeloid cells. Cell Rep..

[115] Vazquez C., Horner S.M. (2015). MAVS coordination of antiviral innate immunity. J. Virol..

[116] Goubau D., Deddouche S., Reis e Sousa C. (2013). Cytosolic sensing of viruses. Immunity.

[117] Chow K.T., Gale M., Loo Y.-M. (2018). RIG-I and other RNA sensors in antiviral immunity. Annu. Rev. Immunol..

[118] Hou F., Sun L., Zheng H., Skaug B., Jiang Q.-X., Chen Z.J. (2011). MAVS forms functional prion-like aggregates to activate and propagate antiviral innate immune response. Cell.

[119] Seth R.B., Sun L., Ea C.-K., Chen Z.J. (2005). Identification and characterization of MAVS, a mitochondrial antiviral signaling protein that activates NF-kappaB and IRF 3. Cell.

[120] Dixit E., Boulant S., Zhang Y., Lee A.S.Y., Odendall C., Shum B., Hacohen N., Chen Z.J., Whelan S.P., Fransen M. (2010). Peroxisomes are signaling platforms for antiviral innate immunity. Cell.

[121] Meylan E., Curran J., Hofmann K., Moradpour D., Binder M., Bartenschlager R., Tschopp J. (2005). Cardif is an adaptor protein in the RIG-I antiviral pathway and is targeted by hepatitis C virus. Nature.

[122] Li X.-D., Sun L., Seth R.B., Pineda G., Chen Z.J. (2005). Hepatitis C virus protease NS3/4A cleaves mitochondrial antiviral signaling protein off the mitochondria to evade innate immunity. Proc. Natl. Acad. Sci. USA.

[123] Horner S.M., Liu H.M., Park H.S., Briley J., Gale M. (2011). Mitochondrial-associated endoplasmic reticulum membranes (MAM) form innate immune synapses and are targeted by hepatitis C virus. Proc. Natl. Acad. Sci. USA.

[124] Yang S., Harding A.T., Sweeney C., Miao D., Swan G., Zhou C., Jiang Z., Fitzgerald K.A., Hammer G., Bergo M.O. (2019). Control of antiviral innate immune response by protein geranylgeranylation. Sci. Adv..

[125] Jacobs J.L., Zhu J., Sarkar S.N., Coyne C.B. (2014). Regulation of mitochondrial antiviral signaling (MAVS) expression and signaling by the mitochondria-associated endoplasmic reticulum membrane (MAM) protein Gp78. J. Biol. Chem..

[126] Cheng J., Liao Y., Zhou L., Peng S., Chen H., Yuan Z. (2016). Amplified RLR signaling activation through an interferon-stimulated gene-endoplasmic reticulum stress-mitochondrial calcium uniporter protein loop. Sci. Rep..

[127] Yasukawa K., Oshiumi H., Takeda M., Ishihara N., Yanagi Y., Seya T., Kawabata S.-i., Koshiba T. (2009). Mitofusin 2 inhibits mitochondrial antiviral signaling. Sci. Signal..

[128] Castanier C., Garcin D., Vazquez A., Arnoult D. (2010). Mitochondrial dynamics regulate the RIG-I-like receptor antiviral pathway. EMBO Rep..

[129] Onoguchi K., Onomoto K., Takamatsu S., Jogi M., Takemura A., Morimoto S., Julkunen I., Namiki H., Yoneyama M., Fujita T. (2010). Virus-infection or 5’ppp-RNA activates antiviral signal through redistribution of IPS-1 mediated by MFN1. PLoS Pathog..

[130] Hwang M.-S., Boulanger J., Howe J.D., Albecka A., Pasche M., Mureşan L., Modis Y. (2019). MAVS polymers smaller than 80 nm induce mitochondrial membrane remodeling and interferon signaling. FEBS J..

[131] Koshiba T., Yasukawa K., Yanagi Y., Kawabata S.-I. (2011). Mitochondrial membrane potential is required for MAVS-mediated antiviral signaling. Sci. Signal..

[132] Missiroli S., Patergnani S., Caroccia N., Pedriali G., Perrone M., Previati M., Wieckowski M.R., Giorgi C. (2018). Mitochondria-associated membranes (MAMs) and inflammation. Cell Death Dis..

[133] Zevini A., Olagnier D., Hiscott J. (2017). Crosstalk between cytoplasmic RIG-I and STING sensing pathways. Trends Immunol..

[134] Ishikawa H., Barber G.N. (2008). STING is an endoplasmic reticulum adaptor that facilitates innate immune signalling. Nature.

[135] Zhong B., Yang Y., Li S., Wang Y.-Y., Li Y., Diao F., Lei C., He X., Zhang L., Tien P. (2008). The adaptor protein MITA links virus-sensing receptors to IRF3 transcription factor activation. Immunity.

[136] Ishikawa H., Ma Z., Barber G.N. (2009). STING regulates intracellular DNA-mediated, type I interferon-dependent innate immunity. Nature.

[137] Franz K.M., Neidermyer W.J., Tan Y.-J., Whelan S.P.J., Kagan J.C. (2018). STING-dependent translation inhibition restricts RNA virus replication. Proc. Natl. Acad. Sci. USA.

[138] Ablasser A., Chen Z.J. (2019). cGAS in action: Expanding roles in immunity and inflammation. Science.

[139] West A.P., Khoury-Hanold W., Staron M., Tal M.C., Pineda C.M., Lang S.M., Bestwick M., Duguay B.A., Raimundo N., MacDuff D.A. (2015). Mitochondrial DNA stress primes the antiviral innate immune response. Nature.

[140] Sliter D.A., Martinez J., Hao L., Chen X., Sun N., Fischer T.D., Burman J.L., Li Y., Zhang Z., Narendra D.P. (2018). Parkin and PINK1 mitigate STING-induced inflammation. Nature.

